# Prevalence and Distribution of Sagittal and Vertical Subtypes in Skeletal Class III Malocclusion: A Retrospective Study across Age and Sex Groups -

**DOI:** 10.31661/gmj.v14iSP1.4020

**Published:** 2025-12-15

**Authors:** Shiva Tavakol Davani, Soodeh Tahmasbi, Mohammad Hassannia Dargah, Ehsan Lakmazaheri, Niusha Solooki, Amirreza Ahmadpour

**Affiliations:** ^1^ Department of Orthodontics, School of Dentistry, Shahid Beheshti University of Medical Sciences, Tehran, Iran; ^2^ Dental Research Center, School of Dentistry, Shahid Beheshti University of Medical Sciences, Tehran, Iran; ^3^ Department of Orthodontics, Faculty of Dentistry, Qazvin University of Medical Sciences, Qazvin, Iran; ^4^ Dental Research Center, School of Dentistry, Shahid Beheshti University of Medical Sciences, Tehran, Iran

**Keywords:** Sagittal Subtypes, Vertical Subtypes, Skeletal Class III Malocclusion

## Abstract

**Background:**

Skeletal Class III malocclusion causing mandibular prognathism, maxillary retrognathism, or a combination of both, has widely varying prevalence by ethnicity. The objective of this research is to investigate the prevalence of contributing factors in its development across different age and sex groups of Iranians.

**Materials and Methods:**

In this study, 233 lateral cephalograms of patients with skeletal Class III malocclusion who had referred our orthodontics center from 2015 to 2024. In the sagittal dimension, the samples were categorized into four groups: retrognathic maxilla, prognathic mandible, combination, and normal. The prevalence of each condition was analyzed across groups. In case of discrepancies between Steiner and McNamara analyses, final sagittal diagnoses were manually determined.

**Results:**

This study examined 233 lateral cephalograms of individuals with skeletal Class III malocclusion, including 101 males (43.3%) and 132 females (56.7%). There was a significant association between the final sagittal relationship diagnosis and the combined age-sex groups (P=0.010), indicating that the distribution of sagittal patterns varies across age and sex subgroups. Mandibular prognathism was the most prevalent condition in all groups except for females aged 7–11, where mandibular prognathism and maxillary retrognathism were equally common. In the vertical dimension, 51.9% of cases had normal facial height. No significant correlation was found between vertical dimension and age or sex (P=0.479). The strongest positive correlation was observed between Sum.o.p and SN.GoMe, as well as Wits and A. NPPD, while the strongest negative correlations involved Jarabak.index with Sum.o.p and SN.GoMe.

**Conclusion:**

The results indicated that mandibular prognathism is the predominant cause of skeletal Class III malocclusion, except in females aged 7–11, where maxillary retrognathism is also prevalent. No discernible pattern was detected in vertical classification among age and sex groups. The research shows the significance of employing various diagnostic methods for a thorough assessment. Subsequent research needed to integrate longitudinal studies and advanced imaging techniques to improve diagnostic precision.

## Introduction

Class III malocclusion is defined as a condition in which the lower teeth or jaw is significantly anterior to the upper teeth or jaw in the sagittal dimension [1]. The causes of this malocclusion can be skeletal, dental, or a combination of the two. Furthermore, even if the individual's skeletal conditions are perfectly normal, this malocclusion could be the result of a functional shift caused by a premature contact [2]. Class III malocclusion is characterized by a complex three-dimensional skeletal imbalance of maxillary and mandibular growth, as well as varying degrees of dentoalveolar and soft tissue compensation [3]. Identifying the etiology of Class III malocclusion (skeletal, dental, or combined) and discerning whether the primary contributor to Class III skeletal malocclusion is the retrognathic maxilla or the prognathic mandible are critical considerations for early intervention and appliance selection [4]. Steiner analysis is preferable for an in-depth evaluation of dental relationships, whereas Wits index is advantageous for a more rapid and straightforward assessment of the condition [5]. The McNamara analysis is a popular orthodontic method for assessing skeletal malalignment and assisting with diagnosis and treatment planning [6]. Sex and age play a significant role in determining normal cephalometric values. As a result, the proper range for each of these indicators in an orthodontically balanced face should be determined based on age and sex [7]. Previous studies focused on the cephalometric characteristics of Class III malocclusion in specific population groups, such as surgical patients [8], children and adolescents [9][10], and military personnel [11]. This focus limits the findings' generalizability to larger age groups and populations. Furthermore, much of the existing research has focused on the anteroposterior relationship between the maxilla and mandible [12][11], with relatively little attention paid to the vertical facial dimension and its role in the various subtypes of Class III malocclusion. Furthermore, previous research has not adequately investigated a comprehensive classification of these subtypes and their distribution across different age and sex groups.

This retrospective descriptive-analytical study aims to analyze the skeletal components of Class III malocclusion and assess the distribution and prevalence of its sagittal and vertical subtypes in order to evaluate skeletal patterns across different age and sex groups, as well as provide a more comprehensive understanding of the skeletal characteristics associated with this condition.

## Materials and Methods

### Study Design, Settings, and Population

This is a retrospective descriptive-analytical study, based on available data from more than 1000 patients aged 7-46 in the Orthodontics Department of Shahid Beheshti Dental School and a private radiology center, from January 1, 2015 to December 31, 2024.

The Ethics Committee of Shahid Beheshti University of Medical Sciences reviewed the current study and approved it under the code IR.SBMU.DRC.REC.1402.065. Owing to the retrospective design of the investigation, all lateral cephalometric radiographs utilized were originally acquired for clinical diagnostic and treatment purposes; consequently, no additional radiation exposure was incurred for research purposes, and analyses were confined to existing archival records. Patient confidentiality was ensured by anonymizing all cephalometric images through removal of personal identifiers prior to analysis, and subsequent procedures were recorded from documentaries exclusively using assigned numerical codes for each case.

To calculate the sample size, the following formula was used to estimate sensitivity and specificity in diagnosing the patient's skeletal pattern detected by the software:


n = \frac{z^{2}_{1-\frac{\alpha}{2}} \times p(1-p)}{d^{2}}

At a 95% confidence level (confidence coefficient z_(1-α/2)=1.96) Assuming P=0.5 to maximize the sample size and an absolute estimation error of d=0.07, the minimum sample size was determined to be 200, with 233 samples selected for enhanced accuracy in this study.

Patients over the age of 7 with class III malocclusion, defined by an ANB angle of less than 1 or Wits' appraisal of less than -1, cephalometric images obtained in maximum intercuspation with diagnostic optical density and contrast, were eligible.

Congenital problems or craniofacial anomalies, a history of orthognathic surgery, a history of orthodontic treatment, blurred cephalometric images or non-standard head position, or pseudo-Class III malocclusion were all exclusion criteria.

### Study Measures

Lateral cephalometric radiographs were acquired in JPEG format. Cephalometric tracing and analysis were performed using Dolphin Imaging software (version 11 of Dolphin Imaging & Management Solutions, Chatsworth, CA, USA). One experienced orthodontist manually identified the landmarks listed in Table-1 on each radiograph. To minimize identification errors and enhance accuracy, a second qualified orthodontist independently reviewed and verified the placement of all landmarks. Any discrepancies were resolved through discussion until consensus was reached. Following landmark verification, the software automatically computed the required linear and angular measurements for the selected analyses.

McNamara analysis employs linear measurements to evaluate sagittal jaw relationships (effective midface and mandibular lengths, maxillomandibular differential) and vertical dimensions relative to cranial base references, making it particularly useful for orthognathic surgery planning. Steiner analysis utilizes angular measurements (SNA, SNB, ANB) and planes to assess skeletal and dental relationships, facilitating evaluation of facial harmony. The Jarabak (Björk-Jarabak) analysis focuses on polygonal proportions to classify facial growth patterns (hyperdivergent, hypodivergent) through ratios and posterior angle sums.

Sagittal discrepancies were primarily assessed using parameters from McNamara (A-Nperp, P-Nperp) and Steiner (ANB, Wits appraisal) analyses. Vertical patterns were evaluated via Jarabak index, mandibular plane angle, and sum of posterior angles.

All computed values were exported and organized in a Microsoft Excel spreadsheet for classification and statistical analysis. Custom Excel formulas were incorporated to allow flexible adjustment of normative reference values for each parameter.

To classify sagittal and vertical skeletal relationships, population-specific normative data for Iranian adults were applied, derived from established local study on subjects with normal occlusion [7], as shown in Table-2.

### Study Outcome Measure

**Figure-1:**
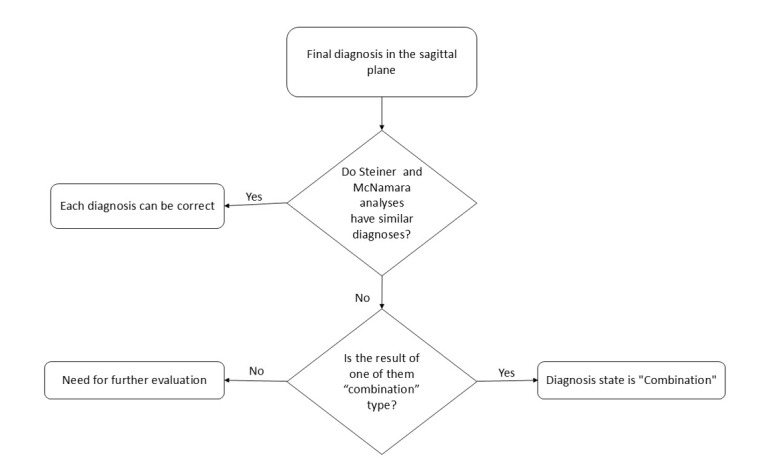
Figure 1. Flowchart of the method for determining the final diagnosis in the sagittal dimension

To detect the culprit jaw in the sagittal dimension, the software automatically considered one of the three prognathic, normal and retrognathic states according to the normal values defined for each jaw. These states were considered separately in both the Steiner and McNamara analyses. If the analyses were consistent, the culprit jaw's final state was reported. Otherwise, three of the authors re-examined the item and manually recorded its state. Figure-1 depicts the steps taken to reach the final diagnosis in the sagittal dimension.

**Figure-2:**
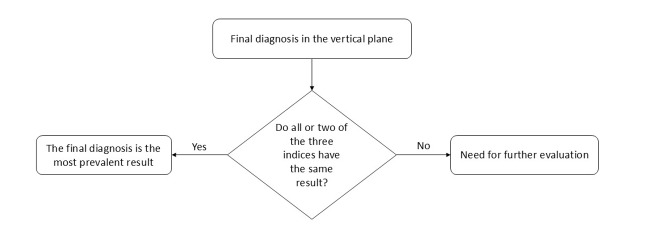
Figure 2. Flowchart of the method for determining the final diagnosis in the vertical dimension

In the vertical dimension, if the indices converged, meaning that all three indices or two of the three indices (mandibular plane angle, sum of posterior, and Jarabak index) indicated the same state, the desired state was automatically reported as one of three forms: Long Face, Normal, or Short Face. Otherwise, the concerned sample was manually re-examined and the appropriate state was recorded. Figure-2 depicts the steps taken to reach the final diagnosis in the vertical dimension.

### Statistical Analysis

SPSS version 23 was used for statistical analysis. In this study, the Pearson chi-square test and Fisher's exact test were used to statistically examine the relationship between qualitative data, including the results of Steiner and McNamara analyses and the final diagnosis in the sagittal dimension, and age and sex categories. The Shapiro-Wilk test was used to determine the normality of the distribution of quantitative variables in the correlation analysis. The Pearson correlation coefficient was also applied to investigate the relationship between quantitative variables.

## Results

**Table:** Table1. Cephalometric Landmarks and Measurements used for Hard Tissue Analysis in Lateral Cephalograms

**Row**	**Landmark/Measurement**	**Definition**
1	Point A (A)	The innermost point on the maxillary contour between anterior nasal spine and incisor tooth
2	Point B (B)	The innermost point on the mandibular contour between incisor tooth and bony chin
3	Sella (S)	The midpoint of sella turcica
4	Nasion (N)	The most anterior point of frontonasal suture
5	Occlusal plane	Line passing through maximum contact points of posterior teeth
6	ANB	Angular difference between SNA and SNB
7	Wits appraisal	Linear distance between perpendiculars from A and B onto occlusal plane
8	Frankfort Horizontal (FH) plane	Line connecting anatomic Porion (Po) and Orbitale (Or)
9	Pogonion (Pog)	The most anterior point on chin contour
10	Gonion (Go)	Midpoint of curvature connecting mandibular ramus and body
11	Menton (Me)	Most inferior point of mandibular symphysis
12	Articulare (Ar)	Intersection point of posterior border of mandibular ramus and inferior border of occipital bone
13	Orbitale (Or)	Lowest point on infraorbital margin
14	Porion (Po)	Midpoint of superior margin of external auditory meatus
15	A to N perpendicular to FH (A-Nperp)	Distance from point A to the line perpendicular to the Frankfurt plane from point N
16	Pog to N perpendicular to FH (P-Nperp)	Distance from point Pog to the line perpendicular to the Frankfurt plane from point N
17	Mandibular plane angle	Angle between the mandibular plane (Go-Me) and the anterior cranial base (SN)
18	Jarabak index	The ratio of posterior facial height (S-Go) to anterior facial height (N-Me or equivalent)
19	Sum of posterior angles	Sum of saddle angle (N-S-Ar), articular angle (S-Ar-Go), and gonial angle (Ar-Go-Me)

**Table:** Table2. Normal Numerical Ranges forSN-GoMe, Jarabak Index, and Sum of Posterior Indices, Pog-N Perpendicular, A-N Perpendicular, SNB, and SNA

**Sex**	**Age (years)**	**SN-GoMe (degrees)**	**Jarabak index (%)**	**Sum of posterior (degrees)**	**SNA (degrees)**	**SNB (degrees)**	**A-Nperp** **(millimeters)**	**P-Nperp** **(millimeters)**
**Male**	7-11	28.42-35.32	61.28-67.12	391.22-398.22	75.21- 81.43	74.10- 76.86	(-5.31) - 1.63	(-10.91) - (-2.05)
**Male**	12-17	28.45-34.51	61.72-69.78	390.54-397.14	77.59- 82.49	74.04- 78.72	(-3.28) - 0.84	(-11.17) - (-4.19)
**Male**	At least 18	18.87-32.73	63.63-76.91	381.90-396.18	77.96- 85.92	76.85- 82.03	(-1.71) - 2.75	(-4.99) - 1.79
**Female**	7-11	30.08-35.56	58.86-64.28	393.09-398.79	77.53- 82.59	76.47- 79.25	(-2.97) - 1.65	(-6.43) - 0.51
**Female**	12-17	27.33-34.47	64.37-71.07	390.11-397.41	75.82- 82.58	72.68- 78.44	(-4.17) - 2.15	(-13.90) - (-7.06)
**Female**	At least 18	24.86-34.56	63.75-69.81	387.90-397.74	78.58- 83.56	75.27- 80.83	(-5.07) - 1.97	(-13.36) - 0.08

**Table:** Table3. Frequency Distribution by Age-sex Groups for Final Diagnosis

**Gender**		**Male**			**Female**		**P1**[N1]	**P2**	**P3**
**Age group**	**7-11**	**12-17**	**≥18**	**7-11**	**12-17**	**≥18**			
Total	30	35	36	36	56	40			
**Sagittal Relationship**									
Maxillary Retrognathism	3 (10%)	3 (8.6%)	9 (25%)	15 (41.7%)	6 (10.7%)	7 (17.5%)			
Mandibular Prognathism	23 (76.7%)	24 (68.6%)	20 (55.6%)	15 (41.7%)	37 (66.1%)	21 (52.5%)	0.01	0.056	0.389 / 0.006
Combined	4 (13.3%)	7 (20.0%)	6 (16.7%)	3 (8.3%)	12 (21.4%)	8 (20%)			
Normal Sagittal Relationship	0 (0%)	1 (2.9%)	1 (2.8%)	3 (8.3%)	1 (1.8%)	4 (10%)			
**Vertical Pattern**									
Short Face	3 (10.0%)	7 (20.0%)	2 (5.6%)	7 (19.4%)	6 (10.7%)	4 (10.0%)			
Long Face	9 (20.0%)	11 (21.4%)	10 (27.8%)	13 (26.1%)	23 (41.1%)	17 (42.5%)	0.479	0.624	0.362 / 0.745
Normal	18 (60.0%)	17 (48.6%)	24 (66.7%)	16 (44.4%)	27 (48.2%)	19 (47.5%)			

P1: Pearson’s χ² test of independence between diagnosis and the six combined age-sex groups P2: Pearson’s χ² test of independence between diagnosis and age group (sexes combined) P3: Pearson’s χ² test of independence between diagnosis and age group, performed separately for males

**Figure-3:**
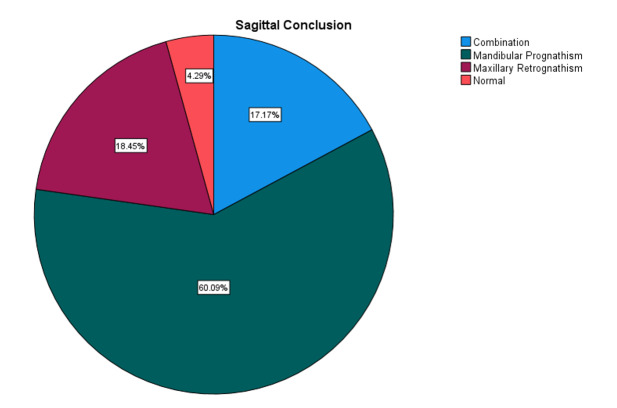
Figure 3. Pie chart of the frequency of different final diagnosis states in the sagittal dimension, in all samples.

**Figure-4:**
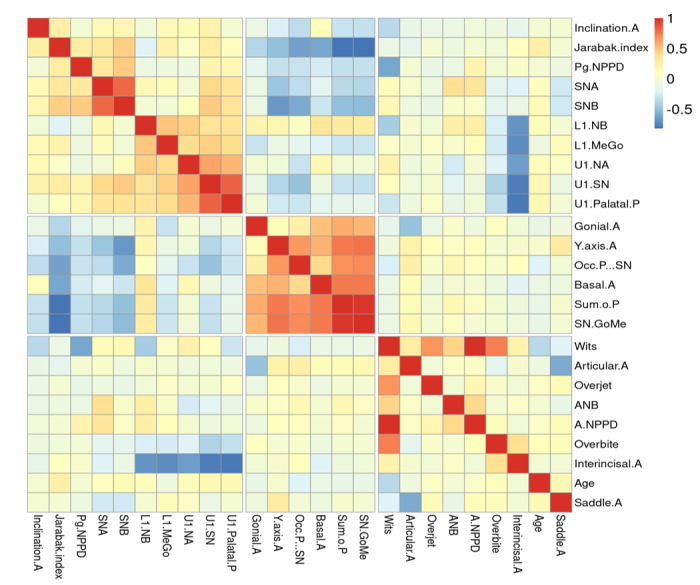
Figure 4 . Correlation between different variables in patients with class III malocclusion; In this diagram, the dark red color represents a strong and direct correlation, while the dark blue color represents a strong but inverse relationship. The pale dots in the figure also indicate the absence of correlation or a small correlation between the relevant data.

In this study, 233 lateral cephalograms were examined based on the inclusion and exclusion criteria. Of the 233 samples with skeletal Class III malocclusion, 101 (43.3%) were male and 132 (56.7%) were female.

In the sagittal dimension, the Steiner analysis identified maxillary retrognathism in 46 cases (19.7%), mandibular prognathism in 133 cases (57.1%), combined maxillary retrognathism and mandibular prognathism in 8 cases (3.4%), and normal sagittal relationships in 46 cases (19.7%).

The McNamara analysis revealed similar patterns, with maxillary retrognathism in 45 cases (19.3%), mandibular prognathism in 134 cases (57.5%), combined discrepancies in 20 cases (8.6%), and normal sagittal relationships in 34 cases (14.6%).

Given that the results of the Steiner and McNamara analyses differed in some cases, all of these samples were re-examined, and the final diagnosis in the sagittal dimension was manually entered. When integrating both analyses for an overall diagnosis, mandibular prognathism remained the most prevalent finding in 140 cases (60.1%), followed by combined discrepancies in 40 cases (17.2%), maxillary retrognathism in 43 cases (18.5%), and normal sagittal dimension in only 10 cases (4.3%). Figure-3 depicts the frequency of each final diagnosis condition in the sagittal dimension as a pie chart. Finaly, 121 (51.9%) samples had normal vertical facial dimensions. Furthermore, 83 (35.6%) and 29 (12.4%) samples had vertical dimensions of long face and short face, respectively.

Statistical analyses using Pearson’s χ² tests revealed a significant association between the final sagittal relationship diagnosis and the combined age-sex groups (χ²=30.58, df=15, P=0.010), indicating that the distribution of sagittal patterns varies across age and sex subgroups. When sexes were combined, the association between sagittal diagnosis and age group was not significant (χ²=12.27, df=6, P=0.056). Subgroup analysis showed no significant age-related differences in males (P=0.389), but there was a highly significant difference in females (P=0.006), showing the higher prevalence of maxillary retrognathism in the 7-11-year-old female group. In contrast, no significant associations were found between vertical facial pattern and age-sex groups (χ²=9.57, df=10, P=0.479), age alone (P=0.624), or age within males (P=0.362) or females (P=0.745), confirming similar distributions of vertical patterns across all groups. Table-3 shows the results of these analyses by age and sex group for final diagnosis. Figure-4 shows the Pearson correlation between cephalometric variables, age, and other variables associated with Class III malocclusion. Age shows very weak correlations with almost all cephalometric variables (mostly pale yellow), indicating minimal influence of age on these measurements within the studied sample.

The most positive association was seen between Sum.o.p and SN.GoMe, as well as between Wits and A.NPPD. However, the strongest negative correlation was found between Jarabak.index and both Sum.o.P and SN.GoMe, as well as the correlation between Interincisal.A and both U1.SN and U1.Palatal.P. All correlations between vertical indices and sagittal indices are negative. The negative correlations are consistently stronger with SNB (mandibular position) than with SNA (maxillary position). Wits shows a noticeably stronger correlation with overbite than with overjet, suggesting the functional occlusal plane affects Wits values.

## Discussion

This retrospective descriptive-analytical study was conducted to analyze the skeletal components of Class III malocclusion and to assess the distribution and prevalence of its sagittal and vertical subtypes in various age and sex groups. In the current study, the samples were divided into six age-sex groups, and the culprit condition causing skeletal Class III malocclusion was investigated separately in each subgroup. There was a significant relationship between the results of the Steiner, McNamara analysis and the final diagnosis in the sagittal dimension with age and sex groups. The most common cause of skeletal Class III malocclusion was mandibular prognathism. Furthermore, the most common condition in all age-sex groups, with the exception of the female group aged 7 to 11 years, was mandibular prognathism.

In our study, the most common cause of skeletal Class III malocclusion, regardless of age or sex, was mandibular prognathism-normal maxilla condition. The vertical dimension diagnosis in this condition was mostly normal face. A similar result was obtained in the group of 17 years and older, which was consistent with the findings of Kodrian's et al. [14] study in the adult Iranian population in the horizontal dimension. In the vertical dimension, the majority of the samples in Kodrian's et al. [14] study were of normal facial height, which was consistent with the findings of our study.

Furthermore, except for the 7-11 year old female group, the highest frequency was associated with the mandibular prognathism-normal maxilla condition, which was consistent with the findings of THIRUMAGAL et al. [15], who also considered age groups.

Our study and Li's study [16] both indicated mandibular prognathism as a main feature of Class III malocclusion; however, there are discrepancies in classification and vertical assessment. While our study found that mandibular prognathism was the most common condition, except in females aged 7-11, where maxillary retrognathism was also common, this study used cluster analysis to divide patients into four subtypes based on skeletal discrepancies, incisor inclinations, and vertical dimensions.

The differences in our findings relative to some prior research [10][17][18][19][20] earlier studies that reported maxillary deficiency in the majority of Class III individuals they evaluated, can be ascribed to differences in diagnostic methods, sample selection criteria, statistical methods, and population characteristics. In contrast, our study employed both Steiner and McNamara analyses, with final sagittal diagnoses conducted manually, potentially resulting in a more specific classification that highlights mandibular prognathism as the primary characteristic.

Ellis [2] found that in a group of American adults, regardless of sex, the combination condition (maxillary retrognathism-mandibular prognathism) had the highest prevalence (30.1%) Furthermore, the prevalence of the retrognathic maxilla-normal mandible group and the normal maxilla-prognathic mandible group was nearly equal (19.5% and 19.2%, respectively), which contradicts our findings in the 17-year-old and older group. Furthermore, the Ellis [2] study reported the highest frequency of diagnosis in the vertical dimension for adults as long face, whereas our study found the highest frequency in the adult group as normal facial height.

The correlation of angles and distances measured on lateral cephalograms resulted in a 25x25 matrix with a complex interpretation. Figure-4 depicts the matrix as a heat map. The Wits index showed a strong correlation (positive and negative) with the McNamara A-Nperp and Pog-Nperp indices in the sagittal dimension. This correlation was stronger than the correlation with the Steiner SNA and SNB values. Furthermore, the Wits index was more strongly correlated with overbite than with overjet, indicating that the functional occlusal plane played a role in the Wits index.

Among the correlations reported in our study, the correlation of Wits index with ANB in patients with any occlusal condition was previously investigated in a study by Al-Jabaa [21], who found a significant positive correlation between these two indices. Our study found a positive correlation between these two indices in patients with skeletal Class III malocclusion, but it was not statistically significant.

Figure-4 depicts the correlation between overjet and sagittal analysis indices of patients (such as Wits, Steiner, and McNamara). Among these indices, the correlation between overjet and Wits index was the strongest, which can be attributed to the influence of teeth on the numbers related to both indices.

Figure-4 depicts the correlation of the indices related to the vertical dimension in its center. The Sum of posterior angles and the SN-GoMe angle had the strongest positive correlation among the vertical indices measured in our study. Both of these indices had a significant negative correlation with the Jarabak index.

The correlation of vertical dimension indices with sagittal dimension indices is depicted in the middle part of Figure-4, on the left. All of these correlations are negative, and their correlation value with SNB is greater than SNA.

As shown in the middle section of Figure-4 on the left, the Y-axis angle has a significant negative correlation with SNB and SNA, with SNB having a higher correlation value than SNA.

The present study's heat map shared significant similarities with the minimum spanning tree drawn from weighted graphs related to the correlation between cephalometric data in Jeong et al.'s [24] study. In our study, color intensity is used to show correlation intensity rather than line thickness, and the data with the highest correlation are plotted as separate rectangular areas rather than arranged in a tree.

## Conclusion

The findings of this study indicate that mandibular prognathism is the primary factor in skeletal Class III malocclusion across most age and sex groups, with the exception of females aged 7-11, where both mandibular prognathism and maxillary retrognathism were prevalent equally. In terms of vertical classification, the majority of cases fell within a normal range, showing no significant association with age or sex. The results of this study suggest that there is a considerable alignment between Wits and McNamara analyses, emphasizing the advantage of utilizing multiple diagnostic approaches for a more comprehensive evaluation. This study found variability in sagittal discrepancies based on age and sex; however, no discernible pattern was identified in the vertical dimension. These results underline the importance of thorough skeletal analysis in planning treatments. Future investigations could benefit from long-term studies and the incorporation of advanced imaging technologies to improve the precision of diagnoses.

## Conflict of Interest

None.

## References

[R1] Kozanecka A, Sarul M, Kawala B, AntoszewskaSmith J (2016). Objectification of Orthodontic Treatment Needs: Does the
Classification of Malocclusions or a History of Orthodontic Treatment Matter. Adv Clin Exp Med.

[R2] Ellis E, McNamara JA (1984). Components of adult Class III malocclusion. J Oral Maxillofac Surg.

[R3] Sanborn RT (1955). Differences Between the Facial Skeletal Patterns Of Class III
Malocclusion and Normal Occlusion*. The Angle Orthodontist.

[R4] Azamian Z, Shirban F (2016). Treatment Options for Class III Malocclusion in Growing Patients
with Emphasis on Maxillary Protraction. Scientifica.

[R5] William Proffit, Brent Larson, David Sarver (2018).

[R6] StornioloSouza JM, Seminario MP, PinzanVercelino CRM, Pinzan A, Janson G (2021). McNamara analysis cephalometric parameters in WhiteBrazilians,
Japanese and JapaneseBrazilians with normal occlusion. Dental Press J Orthod.

[R7] Esmaeili S, Mohammadi NM, Khosravani S, Eslamian L, Motamedian SR (2023). Evaluation of facial profile characteristics of aesthetically
pleasing Iranian faces. J World Fed Orthod.

[R8] Baik HS, Han HK, Kim DJ, Proffit WR (2000). Cephalometric characteristics of Korean Class III surgical
patients and their relationship to plans for surgical treatment. Int J Adult Orthodon Orthognath Surg.

[R9] McNamara JA (1984). A method of cephalometric evaluation. American Journal of Orthodontics.

[R10] Mouakeh M (2001). Cephalometric evaluation of craniofacial pattern of syrian
children with class III malocclusion. American Journal of Orthodontics and Dentofacial Orthopedics.

[R11] Staudt CB, Kiliaridis S (2009). Different skeletal types underlying Class III malocclusion in a
random population. Am J Orthod Dentofacial Orthop.

[R12] Spalj S, Mestrovic S, Lapter Varga, Slaj M (2008). Skeletal components of class III malocclusions and compensation
mechanisms. J Oral Rehabil.

[R13] Paixão MB, Sobral MC, Vogel CJ, Araujo TM (2010). Comparative study between manual and digital cephalometric
tracing using Dolphin Imaging software with lateral radiographs. Dental Press Journal of Orthodontics.

[R14] Koodaryan R, Rafighi A, Hafezeqoran A (2009). Components of Adult Class III Malocclusion in an Iranian
Population. J Dent Res Dent Clin Dent Prospects.

[R15] K T, Naveen K, Prasanna Arvind (2022). PREVALENCE OF CLASS III SKELETAL PATTERN AMONG PATIENTS SEEKING
ORTHODONTIC TREATMENT. Journal of Pharmaceutical Negative Results.

[R16] Li C, Cai Y, Chen S, Chen F (2016). Classification and characterization of class III malocclusion in
Chinese individuals. Head Face Med.

[R17] Ramezanzadeh B, Pousti M, Bagheri M (2007). Cephalometric Evaluation of Dentofacial Features of Class III
Malocclusion in Adults of Mashhad, Iran. J Dent Res Dent Clin Dent Prospects.

[R18] Toms AP (1989). Class III malocclusion: a cephalometric study of Saudi Arabians. Br J Orthod.

[R19] Kao CT, Chen FM, Lin TY, Huang TH (1995). The craniofacial morphologic structures of the adult with Class
III malocclusion. Int J Adult Orthodon Orthognath Surg.

[R20] Ishii N, Deguchi T, Hunt NP (2002). Craniofacial differences between Japanese and British Caucasian
females with a skeletal Class III malocclusion. Eur J Orthod.

[R21] Aljabaa A, Aldrees A (2014). ANB, Wits And Molar Relationship, Do They Correlate In
Orthodontic Patients. Dentistry.

[R22] Jabbar A, Mahmood A (2012). Correlation of overjet, ANB and wits appraisal for assessment of
sagittal skeletal relationship. Pakistan Orthodontic Journal.

[R23] Ardani I, Willyanti I, Narmada IB (2018). Correlation between vertical components and skeletal Class II
malocclusion in ethnic Javanese. Clin Cosmet Investig Dent.

[R24] Jeong S, Kim S, Lim SH, Yu SK (2024). A study of correlations between cephalometric measurements in
Koreans with normal occlusion by network analysis. Scientific Reports.

